# The Roles of the Ubiquitin–Proteasome System in the Endoplasmic Reticulum Stress Pathway

**DOI:** 10.3390/ijms22041526

**Published:** 2021-02-03

**Authors:** Junyan Qu, Tingting Zou, Zhenghong Lin

**Affiliations:** School of Life Sciences, Chongqing University, Chongqing 401331, China; qujunyan1229@yeah.net (J.Q.); tingtzou1997@163.com (T.Z.)

**Keywords:** endoplasmic reticulum stress (ER stress), UPR, UPS, E3 ubiquitin ligases, deubiquitinases

## Abstract

The endoplasmic reticulum (ER) is a highly dynamic organelle in eukaryotic cells, which is essential for synthesis, processing, sorting of protein and lipid metabolism. However, the cells activate a defense mechanism called endoplasmic reticulum stress (ER stress) response and initiate unfolded protein response (UPR) as the unfolded proteins exceed the folding capacity of the ER due to the environmental influences or increased protein synthesis. ER stress can mediate many cellular processes, including autophagy, apoptosis and senescence. The ubiquitin-proteasome system (UPS) is involved in the degradation of more than 80% of proteins in the cells. Today, increasing numbers of studies have shown that the two important components of UPS, E3 ubiquitin ligases and deubiquitinases (DUBs), are tightly related to ER stress. In this review, we summarized the regulation of the E3 ubiquitin ligases and DUBs in ER stress.

## 1. Introduction

The endoplasmic reticulum (ER) is one of the most important organelles in eukaryotic cells and is the main place for calcium storage, protein synthesis and lipid metabolism [1]. Therefore, the physiological activity and homeostasis of the ER are crucial to cell fate. When affected by hypoxia, aberrant Ca^2+^ regulation, nutritional deficiency, low pH, increased protein synthesis, etc., it will quickly destroy the protein-folding ability of the ER, which triggers a defense mechanism called endoplasmic reticulum stress (ER stress) [2]. To alleviate this situation, the stressed cells activate the unfolded protein response (UPR) to help the ER restore function [3].

The ubiquitin–proteasome system (UPS) is the main pathway of protein degradation in eukaryotic cells [4]. Ubiquitination of the target protein is a reversible post-translational modification, which can occur under the successive actions of E1 ubiquitin-activating enzymes, E2 ubiquitin-conjugating enzymes, and E3 ubiquitin–ligase enzymes [5]. The deubiquitinases (DUBs) catalyze the hydrolysis of anchored ubiquitin from the target protein to maintain the level of ubiquitin in cells [6]. Both the ubiquitination and deubiquitination of the proteins are involved in many signal transduction pathways in the cells.

In recent years, many studies have found that UPS plays a key role in ER stress and the high selectivity of E3 ubiquitin ligases and DUBs to substrates is important for the specificity of UPS. Thereby, we focused on the latest developments of the E3 ubiquitin ligase and DUB in the regulation of ER stress.

## 2. ER Stress and Unfolded Protein Response

### 2.1. ER Stress

ER is a large organelle composed of a lamellar system, which is the synthesis site of proteins in the cells and the central hub of the protein folding, assembly, modification and transport. At the same time, ER is also involved in the regulation of the lipid, carbohydrate, steroid metabolism and calcium ion level [7,8].

More than one-third of the proteins in eukaryotic cells are synthesized in the ER, which is, therefore, a key factor in maintaining the homeostasis of the intracellular protein [9]. Meanwhile, ER is an organelle that senses various stimuli and transmits related signals. When the external condition changes (such as hypoxia, nutrient deficiency, calcium ion depletion, etc.), ER function is disturbed, protein synthesis is interrupted and unfolded proteins and misfolded proteins are accumulated in the ER. This kind of cellular stress is called endoplasmic reticulum stress [10,11]. In order to restore the protein homeostasis and normal function of the ER, the cells relieve ER stress mainly in two ways. On one hand, the unfolded protein response (UPR) will be initiated. The initiation of UPR enhances the expression of ER chaperone genes and ER stress-related genes, reduces protein synthesis and accumulation, improves protein-folding ability, and promotes the processing of unfolded or misfolded proteins [12,13]. Misfolded proteins, on the other hand, are cleared by the endoplasmic reticulum-associated degradation pathway (ERAD), which transports the folded protein substrate out of the ER by retro-translocation and is further degraded by the ubiquitin-proteasome system [14]. However, when the ER stress lasts too long, or the protein load in the ER far exceeds the folding capacity of the ER, the original phenotype will not be restored, resulting in cell dysfunction and ultimate cell death [15,16].

Increasing numbers of studies showed that ER stress and UPR are becoming key factors in many diseases. ER stress is caused by external stimuli (such as hypoxia, nutrient deficiency) or intracellular damage (such as carcinogenic mutations), so it is often associated with autophagy, hypoxia signaling, mitochondrial biogenesis or reactive oxygen species (ROS) reactions [17,18], resulting in the occurrence of many human diseases including cancer, diabetes, neurodegeneration, ischemia and infectious diseases [19]. The relationship between ER stress and tumor occurrence has been a research hotspot. ER stress not only can induce apoptosis of tumor cells but also can promote cell survival and lead to drug resistance. For instance, the IRE1 (inositol-requiring enzyme 1) signaling pathway induces the expression of the pro-angiogenic factor VEGF, which may be one of the mechanisms of UPR by which promotes the growth of solid tumors [20]. However, in gastric cancer cells, cell death can be induced by activating the IRE1-JNK-CHOP (C/EBP homologous protein) [21]. The regulation of the ER stress on cell fate is multilevel and multilink and depends on different stress conditions. Therefore, a clearer understanding of the complexity of the ER stress and unfolded protein response, as well as various potential molecular mechanisms, is conducive to the discovery of new drug targets and new intervention strategies for therapy [22,23,24,25].

### 2.2. Unfolded Protein Response

Cells are able to sense the accumulation of the misfolded or unfolded proteins in the ER, activate the downstream effectors to restore ER protein homeostasis, and mediate this regulatory signaling pathway, collectively known as the unfolded protein response (UPR) [13].

In the eukaryotes, UPR is mainly mediated by three ER transmembrane sensors, protein kinase R-like ER kinase (PERK), activating transcription factor 6 (ATF6), and inositol-requiring enzyme 1 (IRE1). Under normal conditions, these transmembrane proteins bind to the ER molecular chaperone immunoglobulin-binding protein (BIP, also known as GRP78 or HSPA5) and are inactive. When the ER homeostasis is disturbed, a large number of unfolded proteins aggregate, which drives GRP78 to dissociate from PERK, ATF6, and IRE1 and bind to unfolded proteins. After dissociation from GRP78, PERK, ATF6 and IRE1 are activated, respectively, and initiate the transduction of three major signaling pathways [10,26]. Whether the UPR response can restore ER homeostasis depends on the intensity and duration of the stimulus. Programmed cell death is initiated if the UPR signal is insufficient to restore and maintain ER homeostasis due to excessive or prolonged stimulation [16].

#### 2.2.1. IRE1 Pathway

IRE1 comprises two subtypes, IRE1α and IRE1β, and most mammalian UPR reactions are regulated by IRE1α [27]. After being activated, IRE1α first undergoes phosphorylation and forms a dimer. The activated IRE1α can cut the mRNA encoding X-box binding protein 1 (XBP1) to form new transcripts and translate into active transcription factors XBP1s (spliced XBP1, XBP1s). XBP1s transcribes after reaching the nucleus, and it upregulates the expression of the genes related to the ER stress [28]. Simultaneously, p-IRE1α promotes mRNA degradation through the regulated IRE1-dependent decay (RIDD) process, slows down the synthesis of new polypeptide chains, and reduces ER pressure [29]. Importantly, p-IRE1α can also induce apoptosis through the JNK pathway activated by TRAF2 and the signal pathway mediated by caspase-12 [30].

#### 2.2.2. PERK Pathway

The activation process of PERK is similar to IRE1α. When PERK senses ER stress, its cytoplasmic region undergoes dimerization and autophosphorylation and then induces phosphorylation of its downstream protein eukaryotic translation initiation factor 2α (eIF2α), which inhibits the function of its translation initiation factor, thereby inhibiting protein synthesis and reducing the load on the ER [31]. Despite the reduction in translation, p-eIF2 can promote the synthesis of the transcription factor ATF4. After the synthesis, ATF4 translocates into the nucleus. As a transcription factor, ATF4 can upregulate the ER chaperones and participate in the transcriptional expression of the amino acid transporters, which is conducive to the restoration of ER homeostasis [32].

In addition, if the ER is under continuous stress, ATF4 will induce the expression of GADD153/CHOP, and CHOP will initiate the proapoptotic program to induce cell death [33]. Moreover, CHOP can also interact with ATF4 to bind to the promoter region of the genes encoding proteins, thus increasing protein synthesis, which can directly lead to oxidative stress and cell death [34,35].

#### 2.2.3. ATF6 Pathway

After the dissociation of ATF6 from GRP78, it transports from the ER to the Golgi apparatus, where it will be cleaved and activated. The activated ATF6 enters the nucleus and relieves ER stress from multiple levels by initiating the transcription of a series of genes [26]. ATF6 can also induce the expression of XBP1 mRNA and work together with IRE1 to regulate the activity of XBP1. In addition, PERK can promote the synthesis of ATF6, and ATF4 facilitates the trafficking of ATF6 from the ER to the Golgi by inducing the expression of related transporter genes [36,37,38]. Therefore, the three signaling pathways of UPR are interconnected and mutually regulated to jointly maintain the endoplasmic reticulum homeostasis.

## 3. Ubiquitin-Proteasome System

**Figure 1:**
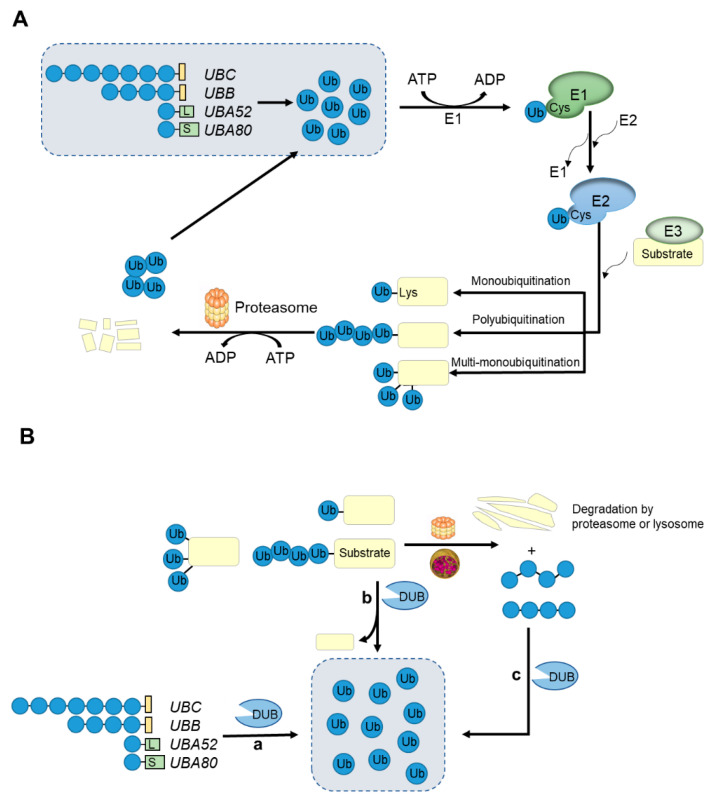
An overview of the ubiquitin-proteasome system and the roles of deubiquitinases (DUBs). (**A**) Ubiquitin is conjugated to substrate proteins by the ATP-dependent successive actions of E1, E2, E3 enzymes that result in target proteins degrading in the 26S proteasome. Proteins can be modified by a single ubiquitin at one or multiple sites or by ubiquitin chains with different lengths and linked sites. (**B**) Ubiquitination is a reversible modification, which is performed by the deubiquitinases. Deubiquitinases (DUBs) can recycle ubiquitin in three ways to maintain the ubiquitin level in cells: (**a**) generate monomeric ubiquitin from precursor proteins encoded by four genes; (**b**) specifically remove ubiquitin or ubiquitin chains from substrate proteins; (**c**) recycle ubiquitin from proteins degraded by proteasomes or lysosomes.

The ubiquitin-proteasome system (UPS) is a protein degradation system that is highly selective and depends on adenosine triphosphate (ATP) for energy supply in eukaryotic cells. About 80% of proteins are degraded by this system, and the rest rely on the autophagy–lysosomal pathway (ALP) for degradation. UPS is composed of ubiquitin, E1 ubiquitin-activating enzymes, E2 ubiquitin-conjugating enzymes, E3 ubiquitin–ligase enzymes, 26S proteasome and DUBs [4]. Ubiquitin is a highly conserved small protein found in all eukaryotes, consisting of 76 amino acid residues [39,40]. Ubiquitination was discovered in 1980, and it is a complex post-translational modification (PTM) of protein [41]. Initially, this modification is mainly involved in the degradation of the proteins. In recent years, an increasing number of studies have shown that ubiquitination also plays an important role in cell cycle regulation, DNA damage, protein localization and activity regulation, protein interaction, apoptosis, autophagy, and occurrence of cancer and neurodegenerative diseases [42,43,44,45,46,47,48,49,50]. Ubiquitination involves a series of successive enzymatic reactions. The glycine (Gly) residue at the *C*-terminus of the ubiquitin can be covalently linked to the lysine (Lys) residue of the target protein under the successive actions of three kinds of ubiquitinases: E1 ubiquitin-activating enzymes, E2 ubiquitin-conjugating enzymes and E3 ubiquitin–ligase enzymes [51,52,53]. Ubiquitination modification mainly involves three processes: (1) activation of ubiquitin molecules. In the presence of ATP, the cysteine (Cys) residue of the E1 forms a thioester bond with the Gly residue at the C-terminus of the ubiquitin, thus activating the free ubiquitin molecule; (2) Ubiquitin is then transferred to the E2 enzyme. Ubiquitin linked to E1 then forms a thioester bond with the Cys residue in the active site of the E2 enzyme, which is transferred to E2 enzyme; (3) Transfer of ubiquitin to the substrate protein. Under the action of the E3 enzyme, the Gly residue of ubiquitin and Lys residue of substrate protein forms an isopeptide bond and are transferred to the substrate protein, and finally form the ubiquitinated substrate [40,54,55] (Figure 1A). In rare cases, the *N*-terminus of the target protein can also be ubiquitinated [56,57,58,59,60]. Normally, ubiquitination can be divided into three types: monoubiquitination, multi-monoubiquitination and polyubiquitination [41]. Monoubiquitination involves a single Lys residue of the substrate attached by a single ubiquitin molecule, while multi-monoubiquitination involves a single ubiquitin couple on multiple Lys residues. Polyubiquitination is the modification of proteins by the ubiquitin chains [41,61]. Ubiquitin can be conjugated by another ubiquitin through any of its seven Lys residues (K6, K11, K27, K29, K33, K48, and K63) or, alternatively, Met1 [62]. Generally, polyubiquitin chains formed at K11 or K48 sites mediate the recognition and degradation of ubiquitinated substrate proteins by 26S proteasome [63]. Polyubiquitin chains linked to the k63 site play an important role in regulating cellular signal transduction, such as the IKK-NF-κB pathway [64]. In addition, monoubiquitination has been shown to be associated with the endocytosis of plasma membrane receptors [61,65]. In the whole ubiquitination process, the most critical step is that the E3 enzyme specifically recognizes the target protein, which is why there are far more types of E3 enzymes than E1 and E2 enzymes [66]. According to the domain characteristics of E3 ligase and the mechanism of transporting ubiquitin, it can be divided into three categories: homogeneous to E6-associated protein C-terminus (HECT), really interesting new gene (RING) and RING-in-between-RING (RBR) [67].

The ubiquitination of proteins is similar to other post-translational modifications such as phosphorylation and acetylation. They are all reversible modifications that can alter the activity, longevity, intracellular distribution and protein interactions of the target proteins [68,69]. The deubiquitination process is carried out by seven deubiquitinases (DUBs) families (MINDY and ZUFSP were recently discovered) [6,52]. DUBs specifically detach ubiquitin from substrate proteins by hydrolyzing ester bonds, peptide bonds or isopeptide bonds at the C-terminus of the ubiquitin. DUBs can release monomer ubiquitin from ubiquitin precursor protein and recover ubiquitin level from ubiquitinated substrate protein and protein degraded by proteasomes or lysosomes [6] (Figure 1B). Therefore, DUBs are important regulators of the ubiquitin system and participate in a variety of cellular regulatory processes, such as protein sorting and transporting, and regulate Wnt and TGF-β signaling pathways [70,71,72,73]. It is speculated that the human genome encodes nearly 100 DUBs. It can be further divided into seven sub-families, USPs/UCHs/OTUs/MJDs/JAMM/MINDY/ZUFSP, and six (except JAMNs) belong to Cys proteases, while the JAMN family is composed of zinc-dependent metalloproteases [6]. The first five families have been widely studied and reported. Each member of the newly discovered MINDY family has the activity of hydrolyzing K48-linked polyubiquitin chains [74,75]. However, only one ZUFSP family has been identified, which shows high selectivity in the hydrolysis of K63-linked polyubiquitin chains. In addition, ZUFSP plays an important role in maintaining genomic stability, preventing spontaneous DNA damage and promoting cell survival in response to exogenous DNA damage [76,77].

In recent years, increasing studies have found that UPS plays an important role in the upstream or downstream of endoplasmic reticulum stress, and the high selectivity of E3 enzymes and DUBs to substrates is an important reason for the specificity of UPS. Therefore, we focused on the latest developments in the study of E3 ligase and DUB in endoplasmic reticulum stress.

## 4. Role of E3 Ubiquitin Ligase in the Regulation of ER Stress

The dynamic regulation of post-translational modification of protein during ER stress is particularly important. Some core proteins of the ER stress can be ubiquitinated and then degraded by 26S proteasome, thereby affecting the progression of the ER stress. There are also some E3 ubiquitin ligases indirectly regulating the process of ER stress through the ubiquitination of proteins related to ER stress (Table 1).

### 4.1. Regulation of the Core Components of ER Stress

#### 4.1.1. MITOL Regulates IRE1α

**Figure 2:**
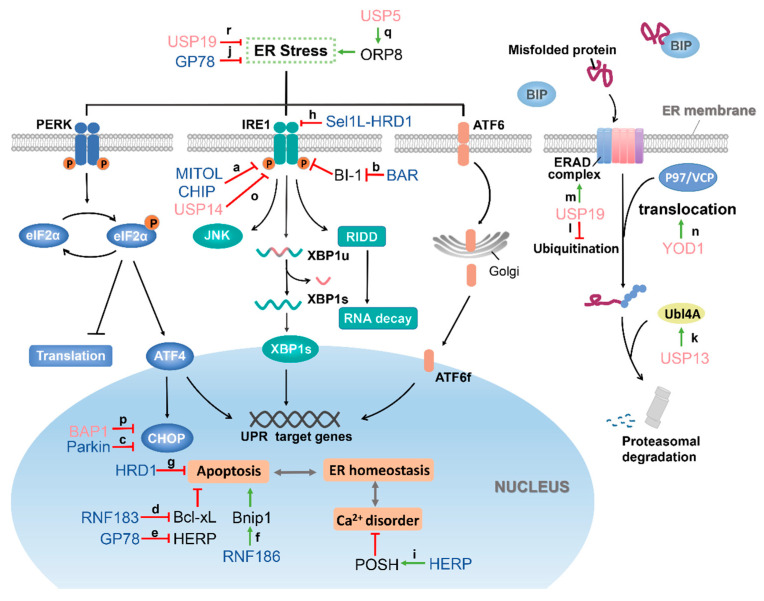
The functional roles of E3 ubiquitin ligases (blue) and DUBs (pink) in the ER stress and ERAD signaling pathway. Once the unfolded protein is overloaded, the three signaling sensor proteins PERK (blue), IRE1 (green), and ATF6 (orange) that reside on the ER membrane are activated, and the activated PERK phosphorylates the eukaryotic translation initiation factor 2α (eIF2α), inhibiting global translation, reducing ER protein-load while also increasing transcription factor ATF4. Activated IRE1 triggers XBP1 mRNA splicing to initiate the synthesis of the transcription factor XBP1. It also enhances mRNA degradation, reduces the total protein content of the ER, and activates JNK and other cellular pathways. ATF6 is translocated into Golgi in response to ER stress and is cleaved to release the transcription factor ATF6f. These transcription factors, in turn, initiate a cascade of signals that relieve ER stress and restore homeostasis or induce cell death pathways. At the same time, the misfolded protein is reverse-transported to the cytoplasm with the assistance of the ERAD complex and the P97/VCP (valosin-containing protein) complex to complete the proteasome degradation. (**a**) The ubiquitination of IRE1α by mitochondrial ubiquitin ligase (MITOL) and carboxy-terminus of Hsc70-interacting protein (CHIP) inhibits its kinase activity, allowing for the suppression of ER stress. (**b**) BI-1 can interact with IRE1α and regulate its endonuclease activity. However, BI-1 is degraded by ubiquitination by bifunctional apoptosis regulator (BAR), leading to the release of the inhibitory effect of IRE1α, thereby promoting ER stress. (**c**) CHOP is violently induced when ER stress occurs, and overexpression of CHOP can lead to cell apoptosis. CHOP is ubiquitinated by Parkin to promote its degradation, which prevents ER stress-induced cell death. (**d**) Bcl-xL is an antiapoptotic protein and belongs to one of the Bcl-2 family members. RNF183 protein increases during ER stress occurs and ubiquitinates Bcl-xL to promote its degradation, leading to increased ER stress-induced apoptosis. (**e**) HERP (homocysteine-induced endoplasmic reticulum protein) is a negative regulator of ER stress, and it can protect cells from apoptosis mediated by ER stress. GP78 can interact with HERP, causing HERP to be ubiquitinated and degraded, thereby starting the process of ER stress recovery. (**f**) Bnip1 is a proapoptotic protein. RNF186 overexpression promotes Bnip1 ubiquitination and transfer to mitochondria but does not affect its protein level. Poly-ubiquitinated BNip1 amplifies RNF186-induced apoptosis signaling of ER stress. (**g**) HRD1 expression is upregulated by ER stress, and HRD1 rescues cells from ER stress-induced apoptosis. (**h**) Sel1L-HRD1 protein complex can recognize, ubiquitinate IRE1α, and mediate its degradation, restraining IRE1α signaling and reduces IRE1α-associated inflammation. (**i**) HERP also plays a vital role in maintaining calcium homeostasis during ER stress. POSH ubiquitinates HERP and promotes the redistribution of HERP from TGN to the ER, thereby regulating calcium homeostasis by increasing the level of HERP in the ER. (**j**) GP78 protects against ER stress in *zebrafish* liver, but the specific protective mechanism is not yet clear. (**k**) USP13 stabilizes UBL4A by deubiquitination, which is a functional component of ERAD, thus allowing proteasome degradation to proceed normally. (**l**) USP19 blocks the degradation of TCRa and other ERAD substrates through deubiquitination. (**m**) In addition, USP19 can deubiquitinate HRD1, which plays a key role in ERAD, and USP19 promotes ERAD by stabilizing HRD1. (**n**) YOD1 eliminates the Ub chains attached to substrates such as RI332 through deubiquitination and promotes the reverse translocation of substrates from the ER to the cytoplasm. (**o**) USP14 binds to IRE1 and inhibits its phosphorylation, thereby blocking IRE1-mediated cell death. (**p**) BAP1 directly binds to promoters of CHOP and inhibits the unfolded protein response (UPR) and associated apoptotic pathways by reducing its transcription. (**q**) ORP8 is a lipid transfer protein anchored to the ER, which can be deubiquitinated by USP5, causing disturbance of phospholipid transport and resulting in ER stress. (**r**) USP19 can inhibit the UPR and downregulate the expression of CHOP, but its specific target is unclear.

Mitochondrial ubiquitin ligase (MITOL, also known as MARCH5) belongs to membrane-associated RING-CH (MARCH) E3 ligase, which locates in the outer membrane of the mitochondria [93]. MITOL can ubiquitinate the mitochondrial fission factor Drp1 to regulate mitochondrial morphology [94]. In addition, recent studies have shown that MITOL is also importantly related to cell senescence, cell survival, stemness maintenance of embryonic stem cells, mitochondrial quality control, immune response, and disease occurrence [93,95,96,97,98]. The endoplasmic reticulum transmembrane protein IRE1α mediates the IRE1α–XBP1 signaling pathway in UPR. During ER stress, IRE1α directly or indirectly recognizes unfolded proteins, leading to autophosphorylation of IRE1α [99]. Recent studies have found that MITOL plays a key role in maintaining ER homeostasis and can resist ER stress. On one hand, compared with the MITOL CS mutant lacking enzymatic activity (C65/68S), the wild-type MITOL can rescue the apoptosis of MITOL-KO MEF cells, suggesting the enzymatic activity of MITOL contributes to the cell survival under ER stress. On the other hand, MITOL ubiquitinates the K481 of IRE1α and forms a polyubiquitin chain through K63 residue, thereby inhibiting the activity of IRE1α (Figure 2a). Hence, MITOL can limit ER stress-dependent cell apoptosis via the suppression of IRE1α-mediated RIDD activity and JNK phosphorylation and regulate IRE1α-dependent decay (RIDD) and JNK phosphorylation. When MITOL was depleted or IRE1α mutated (K481R), RIDD increased, which led to apoptosis. The same results were obtained in *MITOL* deficient mice [78]. Their results suggested that MITOL can inhibit ER stress-induced apoptosis by ubiquitination of IRE1α.

#### 4.1.2. CHIP Regulates IRE1

The carboxy-terminus of Hsc70-interacting protein (CHIP) is a U-box-type ubiquitin ligase, which plays a critical role in cellular senescence, immune regulation, inflammation regulation, and nerve function regulation. In addition, CHIP is also important in the regulation of ER stress. CHIP can ubiquitinate IRE1 on K545 and K828 in a K63-linked manner. This ubiquitination does not promote the degradation of IRE1 and the transduction of the IRE1-XBP1 signaling pathway but affects the kinase activity of IRE1 [79] (Figure 2a). Under ER stress, the kinase activity of IRE1 is activated, and then TRAF2 is recruited to form a complex [30]. The formation of the IRE1/TRAF2 complex is essential for the activation of a series of signaling pathways. However, in CHIP knockdown or K828R mutant of IRE1 cells, this interaction was completely inhibited. These results suggest that the ubiquitination of IRE1 at K828 is critically important for IRE1/TRAF2 interaction [79]. On the other hand, the author also found that the activation of the IRE1/TRAF2/JNK pathway can antagonize the senescence process under ER stress. In addition, it was pointed in another study that CHIP can ubiquitinate TRAF2 and promote its degradation [100]. However, there is no evidence to indicate whether the ubiquitination of TRAF2 has an effect on the formation of IRE1/TRAF2 complex. In short, CHIP can regulate ER stress-induced cell apoptosis and senescence by ubiquitinating IRE1 to promote the formation of IRE1/TRAF2 protein complex.

#### 4.1.3. HRD1 Regulates IRE1α

Hydroxymethyllutaryl reductase degradation protein 1 (HRD1) is an E3 ubiquitin ligase, which is located to the ER membrane and is related to ERAD. In addition to ubiquitination of substrate proteins, HRD1 can also undergo autoubiquitination, which plays an important role in the reverse translocation of misfolded proteins from ER to the cytoplasm [101,102]. The relationship between HRD1 and ER stress was reported as early as 2002. In yeast, the expression of HRD1 is regulated by IRE1α; in mammals, HRD1 is regulated by IRE1α and ATF6 [103]. A study showed [80] HRD1 in cardiomyocytes is upregulated under ER stress. Knockdown of HRD1 will increase the protein levels of ER stress markers Grp94 and Grp78 and increase the level of misfolded endoplasmic reticulum protein and ER stress. In addition, knockdown of HRD1 vastly increased the expression level of CHOP protein, decreased the viability of cardiomyocytes and increased caspase-dependent apoptosis induced by ER stress. This indicated that endogenous HRD1 could protect cardiomyocytes from apoptosis caused by ER stress (Figure 2g).

The Sel1L-HRD1 protein complex is the most conserved ERAD system in mammals, and Sel1L is its essential component. Many studies have shown that the single-spanning ER membrane protein Sel1L is necessary for the stability and function of HRD1 [104], and loss of Sel1L leads to a profound reduction of HRD1 protein level [105]. In a study by Sun and his team [82], it was found that IRE1α is the endogenous substrate of ERAD mediated by the Sel1L-HRD1 complex. Sel1L-HRD1 interacts with IRE1α under basal conditions and mediates its ubiquitination and degradation. When ER stress happens, it will trigger the separation of IRE1α and ERAD complex, thereby weakening the ubiquitination of IRE1α. After knocking out *Sel1L*, it was found that Xbp1s mRNA levels increased, and TNFα-induced JNK phosphorylation was enhanced. Therefore, Sel1L-HRD1 restrains IRE1α signaling and reduces IRE1α-associated inflammation, at least in part, through ERAD-mediated degradation of IRE1α (Figure 2h). Regulatory T cells (Tregs) play a central role in maintaining immune tolerance and performing immune suppression, and their development is mainly regulated by the expression of the transcription factor FoxP3 [106]. In general, Tregs are unstable under inflammation or other pathological conditions, resulting in weakened inhibitory function. A recent study [81] found that the expression of FoxP3 in Treg decreased during ER stress, which led to Treg instability. Specifical knockout of the *Hrd1* gene in mouse Tregs will trigger the ER stress response and the downregulation of FoxP3 protein levels. This is because the deletion of HRD1 leads to the upregulation of IRE1α. Further studies showed that HRD1 inhibited the IRE1α-p38 pathway. Obviously, the above studies have proved that HRD1 has an inhibitory effect on ER stress.

#### 4.1.4. Parkin Regulates CHOP

Parkin belongs to the RBR E3 ligase family and plays a key role in protecting neurons from various injuries [107,108]. Parkin dysfunction is one of the causes of many neurodegenerative diseases, such as Parkinson’s disease (PD), Alzheimer’s disease (AD), amyotrophic lateral sclerosis (ALS), and Huntington’s disease (HD) [109,110,111,112]. Later, in many studies, it was also proved that Parkin is closely related to mitochondrial homeostasis [113,114]. When ER stress occurs, CHOP is upregulated. However, knockout of Parkin in mice will cause the regulator of the ER stress to be upregulated at both transcription and protein levels. CHOP can serve as a new substrate of Parkin, which can be ubiquitinated by Parkin and targeted for degradation by the 26S proteasome [83] (Figure 2c). After this, the same results were verified in the study of astrocytes. After specifically knocking out Parkin, the expression levels of the ER stress regulator genes ATF6, CHOP, spliced Xbp1, and ATF4 increased [84]. These results proved that lack of Parkin amplifies the ER stress signal.

### 4.2. E3 Ubiquitin Ligases which Positively Regulate the ER Stress

#### 4.2.1. RNF183 Increases ER Stress-Induced Apoptosis by Ubiquitinating Bcl-xL

RNF183 is a membrane protein which locates to the ER membrane and belongs to the RING finger (RNF) protein family. It contains an N-terminal RING-type zinc finger and a C-terminal transmembrane domain [85,115]. It has a typical E3 ubiquitin ligase activity. B-cell lymphoma-extra large (Bcl-xL) is an antiapoptotic protein found in the mitochondrial membrane and belongs to one of the Bcl-2 family members [116]. In the process of prolonged ER stress, RNF183 is upregulated in an IRE1α dependent manner, and overexpression of RNF183 leads to an increase in ER stress-induced apoptosis. Since RNF183 was found to promote cell apoptosis, authors speculated that it might target antiapoptotic factors for degradation. Further research found that RNF183 can interact with Bcl-xL, then ubiquitinating Bcl-xL and promoting its degradation (Figure 2d). The progressive decrease of Bcl-xL level eventually induces the intrinsic apoptotic pathway. After knocking down RNF183, it helps to maintain the level of Bcl-xL and reduce the apoptosis caused by ER stress [85].

#### 4.2.2. RNF186 Regulates ER Stress-Mediated Apoptosis by Interacting with Bnip1

RNF186 and RNF183 mentioned above belong to the RING finger E3 ligase family and are located to the ER membrane [86]. Bnip1 is a proapoptotic protein in the Bcl-2 protein family that only contains the BH3 domain, which exists in the outer mitochondrial membrane and ER [117,118]. It plays an important role in inducing apoptosis, maintaining ER integrity and mitochondrion division [118,119]. It has been proven that RNF186 can participate in the regulation of ER stress-mediated apoptosis. On the other hand, RNF186 is also regulated by the ER stress, and its half-life is prolonged under ER stress. Overexpression of RNF186 in Hela cells, HepG2 cells and mouse primary hepatocytes can upregulate the sensor protein of ER stress IRE1α while knocking down RNF186 can block ER stress [86,120]. Further research found that Bnip1 is a substrate protein of RNF186, which colocalizes and interacts in the ER. RNF186 ubiquitinates Bnip1 and forms a polyubiquitin chain through K29 and K63, which mediates the transport of Bnip1 to mitochondria (Figure 2f). In addition, when ER stress occurs, the ubiquitination level of Bnip1 will be significantly increased due to the upregulation of RNF186. Overexpression of Bnip1 had no effect on ER stress signal, but Bnip1 knockdown could reduce the level of ER stress and apoptosis induced by RNF186. This suggests that Bnip1 may act downstream of RNF186 to amplify the ER stress signal. Another study conducted in mouse primary hepatocytes also proved the relationship between RNF186 and ER stress. Overexpression of adenovirus-mediated RNF186 (Ad-RNF186) in mouse primary hepatocytes can markedly increase the mRNA level of IRE1α but has no effect on PERK and ATF6 expression. In addition, overexpression of RNF186 can also induce an increase in IRE1α and CHOP protein levels. Subsequently, the author also found that overexpression of Ad-RNF186 will promote the expression of inflammatory factors in the ER stress pathway, such as TNFα, IL-6, and MCP1. Obviously, RNF186 not only plays a role in the apoptosis signal pathway mediated by ER stress but also amplifies the inflammatory response signal caused by ER stress.

#### 4.2.3. BAR Interacts with BI-1 to Eliminate the Inhibitory Effect of BI-1 on IRE1α

Bifunctional apoptosis regulator (BAR) is a multi-domain protein, which was originally identified as an inhibitor of the Bax-induced apoptosis pathway, which can bind to Bcl-2 through the SAM domain [121]. BAR is mainly expressed in neurons in the central nervous system and is mainly located in the ER membrane [122]. A later study proved that BAR is also a RING-type E3 ligase [89]. Bax inhibitor-1 (BI-1) is an evolutionarily conserved ER membrane protein that can protect cells from the death stimulus of ER stress. Compared with wild-type mice, the protein levels of IRE1, ATF6, and CHOP in *bi-1* knockout mice (*bi-1*^−/−^) that suffered liver ischemia–reperfusion injury were increased [87]. It was also proved that BI-1 deficient cells showed excessive activation of IRE1α and increased levels of its downstream target XBP-1, and BI-1 could form a protein complex with IRE1α and regulate its endonuclease activity [88]. These results indicated that BI-1 is a negative regulator of ER stress. However, BAR interacts with BI-1 in the ER, induces the ubiquitination of BI-1 and promotes the proteasome degradation of BI-1, thereby eliminating the inhibitory effect of BI-1 on IRE1α (Figure 2b). Similarly, reducing the expression of endogenous BAR can also increase the level of BI-1 protein and specifically inhibit IRE1α signaling transduction [89]. This suggested that the post-translational modification of BI-1 by E3 ligase BAR contributes to the dynamic control of IRE1 signal transduction during ER stress.

### 4.3. E3 Ubiquitin Ligases Which Antagonize ER Stress

#### 4.3.1. GP78 Plays Dual Roles in ER Stress Response

GP78 was originally found in mice and is related to cell migration [123,124]. Later, GP78 was proven to act as a tumor autocrine motility factor receptor (AMFR) to mediate tumor cell invasion and metastasis after binding to AMF [125]. In a study, GP78 was identified as a RING finger E3 ligase largely localized to the ER membrane, which can target endogenous and the heterologous ERAD substrate CD3-δ for proteasome degradation [126]. In the event of ER stress, not only can the half-life of GP78 be prolonged, but also the degradation of CD3-δ mediated by GP78 is also enhanced significantly [127]. Similar results were obtained in *zebrafish* [90]. The GP78 isolated from *zebrafish* was also located in the ER and showed E3 ubiquitin ligase activity. When tunicamycin is used to induce hepatic ER stress in juvenile and adult *zebrafish*, the level of GP78 protein increases. Specifically, knocking out of *gp78* in *zebrafish* embryos or livers does not affect the development of embryos and livers, nor does it cause hepatic ER stress under normal physiological conditions. However, the overexpression of the dominant-negative form of GP78 (GP78-R2M, mutating H354 and H357 in the RING finger domain of GP78 to asparagine) in adult *zebrafish* liver renders adult *zebrafish* more sensitive to tunicamycin-induced ER stress than wild-type *zebrafish*. Further studies found that the sensitization to ER stress is accompanied by upregulation of SREBP (sterol response element-binding protein) target proteins, which revealed that GP78 might regulate hepatic lipid homeostasis by activating the SREBP pathway. These suggested that GP78 plays a key role in regulating hepatic ER stress and lipid metabolism (Figure 2j). In another study, it was found that the Ube2g2-GP78 (E2–E3) complex can mediate HERP (homocysteine-induced endoplasmic reticulum protein) ubiquitination and proteasome degradation after ER stress [91]. HERP is an ER membrane protein that is strongly induced under ER stress [128], and its N-terminus and C-terminus ends face the cytoplasm [129]. Studies have shown that upregulation of HERP can protect cells from apoptosis induced by ER stress [130,131,132,133]. The coupling of the ubiquitin conjugation to the ER degradation (CUE) of GP78 interacts with the UBL (ubiquitin-like) domain of HERP, causing HERP to be ubiquitinated and degraded, thereby initiating the process of ER stress recovery [91] (Figure 2e). Therefore, GP78 seems to play dual roles in the ER stress response.

#### 4.3.2. POSH

POSH (plenty of SH3s) is an E3 ubiquitin ligase with a RING finger structure at the N-terminus [92]. It was initially identified as a Rac1-binding protein and can act as an activator of the JNK and NF-κB signaling pathways [134]. HERP is a protein with a UBL domain induced by ER stress. Suppression of HERP can enhance the apoptosis induced by ER stress, while overexpression of HERP can maintain the calcium level during ER stress and reduce JNK/C-JUN and caspase12 apoptosis signals activated by ER stress [133,135,136]. In short, HERP is an inhibitor of ER stress. In a study, it was found that POSH can directly interact with HERP to make HERP be ubiquitinated and form a polyubiquitin chain through K63 residue. Ubiquitination of HERP will promote its redistribution from TGN (trans-Golgi network) to the ER, thereby regulating calcium homeostasis by increasing the level of HERP in the ER [92] (Figure 2i). Therefore, POSH can indirectly regulate ER stress by regulating HERP.

## 5. Deubiquitinase and ER Stress

As part of the ubiquitin-proteasome system, except for E3 ubiquitin ligases, deubiquitinases are also involved in the regulation of ER stress. On one hand, a deubiquitinating enzyme, such as YOD1, as an important regulatory factor of ERAD, affects the degradation process of misfolded proteins; on the other hand, deubiquitination modification of UPR-related proteins by some deubiquitinases can also regulate ER stress progression (Table 2).

### 5.1. Deubiquitinases Which Are Involved in ERAD

In ER stress, misfolded proteins are recognized by specific components and translocated from ER to cytoplasm, where they are ubiquitinated and degraded by the proteasome. This pathway is known as the endoplasmic reticulum-associated degradation pathway (ERAD) [14]. The basic process of ERAD consists of substrate recognition, ubiquitination, translocation and proteasome degradation. Ubiquitination is the key to successfully reverse transport of the substrates to be degraded. Therefore, E3 ubiquitin ligase is the core component of the ERAD machinery, connecting multiple endoplasmic reticulum lumen and cytoplasmic junction proteins. The synergy of the E3 ligase with these junction proteins results in the misfolded substrates being translocated to the cytoplasm for effective proteasomal degradation [147,148]. At the same time, DUBs also play a critical role in the regulation of ERAD, usually by deubiquitinating ERAD substrates or stabilizing ERAD components to regulate the degradation of misfolded proteins.

#### 5.1.1. USP13

In ERAD, the ubiquitin ligase GP78 labels misfolded proteins with ubiquitin, and the ubiquitin modification acts as a signal to recruit p97/VCP ATPase for translocation into the cytoplasm for degradation [149,150]. Recent studies have shown that GP78 can ubiquitinate not only ERAD substrates but also the ERAD mechanical protein Ubl4A. However, USP13 has been shown to counteract the ubiquitination of Ubl4A by GP78. Substrates in *USP13*-deficient cells accumulated in the ER and translocation of the cells is inhibited. These data suggested that USP13 prevented GP78-mediated ubiquitination of UBL4A by deubiquitination and stabilized the expression of ERAD mechanical protein Ubl4A, thus playing a positive role in the degradation of misfolded proteins (Figure 2k). Although the two enzymes have opposite activities, their interactions can coordinate and promote ERAD to maintain ER function [137].

#### 5.1.2. USP19

USP19 is an ER transmembrane protease that is anchored at the tail of the ER and is involved in the regulation of ER stress. When ER stress occurs, USP19 is upregulated, and USP19 can save certain substrates that ERAD degrades by binding tightly to its substrates, such as the T-cell receptor A (TCRa), and protecting them from degradation through USP19 deubiquitination [138] (Figure 2l).

In addition, HRD1, which is known to be the E3 ubiquitin ligase for IRE1α, has been shown to be a novel substrate for USP19 [139]. HRD1 is a ubiquitin ligase resident in the ER. ER stress induces its expression at the transcription level and plays an active role in the degradation of ERAD, thereby preventing cell death due to ER stress [103,151]. Studies have shown that changes in USP19 expression can affect the homeostasis of HRD1. USP19 can maintain HRD1 stable expression of HRD1 under stress conditions through deubiquitination, thus playing an active role in ERAD [139] (Figure 2m).

#### 5.1.3. YOD1

YOD1 is a component of a multiprotein complex with P97 as the core, which is involved in the reverse transport of misfolded proteins in the ER [140]. The results showed that the active site mutant of YOD1 (C160S) inhibited the reverse transport of various misfolded proteins, and results showed that the accumulation of RI332 in YOD1 (C160S) cells (without deubiquitination activity) was much higher than that in YOD1 wild-type cells [141].

According to Sanyal S. et al., the translocation and degradation of RI332 need to be ubiquitinated twice: the first ubiquitination occurs in the ER to recruit p97 and other related proteins to complete the transport. Deubiquitination then occurs, and second ubiquitination is followed in the cytoplasm to label the substrate for proteasomal degradation [152]. Thus, the reversal of misfolded protein transport driven by P97 is impeded, possibly because the Ub chain on the relevant transport substrate intermediate prevents substrate movement to the cytoplasm, leading to significant accumulation of misfolded proteins in the ER [141]. They speculated that YOD1 eliminated the obstacles to the attachment of the Ub chain through its deubiquitination activity, enabling the substrate to successfully complete the transport (Figure 2n).

Interestingly, YOD1 also plays a role in the translocation of non-ubiquitinated substrates. Cholera toxin A1 peptide (CTA1) is a recognized non—ubiquitinated ERAD substrate [153]. For poisoned host cells, CTA1 is transported retrogradely from the plasma membrane of intestinal epithelial cells to ER, catalyzing the formation of the CTA1 subunit in ER. CTA1 then disguises itself as a misfolded protein and uses the ERAD machinery to reverse transport to the cytoplasm [154]. Studies have shown that YOD1 negatively regulates the reverse transport of CTA1, and the mechanism of action may be the deubiquitination of some ERAD components that promote reverse transport of the protein rather than substrate ubiquitination. YOD1 can bind to HRD1, and HRD1 as an ER-associated E3 is crucial in the process of reverse transport. It is implied that YOD1 may interfere with the reverse transport of non-ubiquitinated substrates by deubiquitinating HRD1 [155,156].

### 5.2. UPR-Related Deubiquitinases

#### 5.2.1. USP14

USP14 affects the occurrence and development of many diseases, such as Huntington’s disease (HD). Huntington’s disease is caused by mutations in the Huntingtin protein (Htt) that form aggregates in cells [157]. In Htt-mutated cells, ER stress and its related pathways, such as IRE1, were significantly activated, resulting in cytotoxicity and cell death [158,159]. Previous studies have identified that USP14 can directly bind to IRE1α, and the endogenous interaction between USP14 and IRE1α is inhibited by ER stress [142]. Similar phenomena have been observed in HD disease models. USP14 interacts with IRE1α in control cells, but there is less interaction in mutant Htt cells. While overexpression of USP14 inhibited phosphorylation of IRE1α in mutated Htt cells, protecting the cells from toxic injury and inhibiting the activation of caspase-3 (Figure 2o). These results suggest that ER stress-mediated IRE1α activation is part of mutant-Htt toxicity and that USP14 can reverse this by binding to IRE1α [143]. Although it is not clear which specific signaling pathways downstream of IRE1 are affected by USP14, USP14 does regulate ER stress-mediated cell death through IRE1, so USP14 may be a potential target for future treatment of HD and other clustered diseases.

However, USP14 plays a negative role in metabolic diseases such as obesity type 2 diabetes (T2DM). There is a strong correlation between ER stress and impaired glucose homeostasis, and ER stress also plays a vital role in the occurrence and progression of T2DM [160,161]. Multiple ER stress markers are activated in the livers of obese mice and humans [162]. Studies have shown that sustained ER stress upregulates USP14 from the transcription level through ATF4, thus improving the stability and level of CREB-binding protein CBP, enhancing the role of glucagon and hepatic gluconeogenesis. At the same time, liver-specific knockout of *USP14* eliminated the effect of ER stress on glucose metabolism and also improved hyperglycemia and glucose intolerance in obese mice. In conclusion, ER stress induces the upregulation of USP14, which leads to the destruction of glucose homeostasis [163].

#### 5.2.2. BAP1

BRCA1-associated protein 1 (BAP1) is a nuclear deubiquitination (DUB) enzyme that regulates cellular processes in a DUB-dependent manner, including transcription, DNA replication, and DNA double-strand breaks repair. It is considered to be a tumor suppressor [164].

However, recent studies have shown that BAP1 plays a pro-survival role in cell death induced by ER stress. In *BAP1* knockout mice, ER stress was increased, and they were sensitive to treatment [144]. The unfolded protein reaction (UPR) protects cells from the stress of misfolded proteins, which can trigger cell death if the stress is not relieved or eliminated [26]. The key to UPR-mediated cell fate determination is ER stress-related transcription factors, including XBP1, ATF4, ATF3 and CHOP, etc. [165]. Under glucose starvation, BAP1 can directly bind to the promoter of ATF3 and CHOP, inhibit its transcription, and thus inhibit the UPR and cell death induced by the ER stress (Figure 2p). Notably, the pro-survival effect of BAP1 depends on its deubiquitinase activity [144].

#### 5.2.3. Other DUBs

Recently, it has been reported that the interaction between USP5 and oxysterol-binding protein-related protein 8 (ORP8) can induce ER stress. ORP8 is a lipid transfer protein anchored to ER membrane, and its expression is closely related to tumor cell apoptosis [166,167]. USP5 has a deubiquitination effect on ORP8, which promotes the accumulation of ORP8 through deubiquitination and causes the disturbance of phospholipid transport, which indirectly leads to an aggravation of ER stress in colon cancer cells and induces apoptosis [145] (Figure 2q).

In addition, USP19 can affect the differentiation of muscle cells by regulating UPR. ER stress and the upregulation of CHOP can stimulate the formation of muscle fibers [168,169] while regulating the level of USP19 can affect the fusion and expression of myofibrillar protein [170]. The results have shown that USP19 can inhibit the UPR response and weaken CHOP induction, thereby inhibiting muscle cell differentiation (Figure 2r). Re-induction of ER stress in muscle cells overexpressing USP19 can reverse the fusion defect of muscle cells. However, it is unclear whether USP19 directly affects the UPR process [146].

## 6. Discussion and Conclusions

Here, we systematically discussed the regulation of the ER stress by the E3 ubiquitin ligases and DUBs. It seems that sometimes an enzyme has more than one substrate, and a protein can also be modified by multiple enzymes. Interestingly, the effects of ubiquitination are affected by the modification sites and cellular contents. Modification at different sites and different cellular contents may have different signaling outcomes.

Among the studies that have been reported, IRE1α is mostly studied among the three sensor proteins (IRE1α, ATF6, and PERK). While the E3 ubiquitin ligases for ATF6 and PERK have not been found, IRE1α is reported to be regulated by multiple enzymes. It was found in MEF cells that MITOL can directly regulate IRE1α by promoting its K63-linked ubiquitination at K481, thus mediating RIDD activity and JNK phosphorylation to inhibit ER stress-induced cell death. Notably, the stability of IRE1α is not affected. That is also the case for the regulation of IRE1α by CHIP, which catalyzes the ubiquitination of IRE1α at K545 and K828 in a K63-linked manner. Most importantly, the ubiquitination at K828 is essential for the formation of the IRE1/TRAF2 complex but has no effect on IRE1α stability. In addition, IRE1α is also regulated by the ERAD system, Sel1L-HRD1. Sel1L-HRD1 can recognize and ubiquitinate IRE1α, partially restraining IRE1α signal transduction by ubiquitination-mediated degradation.

The signaling transduction events occurring in cells are extremely complex and cellular content dependent. For instance, GP78 plays a dual role in regulating ER stress. It can protect *zebrafish* liver against ER stress, and it can also ubiquitinate HERP and mediate its degradation to restore the ER stress. At the same time, HERP is regulated by POSH. POSH ubiquitinates HERP to redistribute it to the ER, thereby regulating calcium homeostasis during ER stress. These studies proved that the biological events that occur in cells are extremely complex and changeable.

UPS is the most important way of protein degradation, and protein ubiquitination and deubiquitination involve many cellular processes. Due to the deep research of the scientists, we have a clearer understanding of the mechanism of action and regulation of E3 ubiquitin ligases and deubiquitinating enzymes. In recent years, increasing studies have shown that many ubiquitin ligases and DUBs are closely related to autophagy, cell cycle regulation, and signal transduction. However, so far, the regulation of ER stress by ubiquitin ligase and DUB has received relatively less attention. ER stress is activated by excessive accumulation of unfolded or misfolded proteins in cells. It is one of the causes of many human diseases such as neurodegenerative diseases, diabetes, cancer and cardiovascular diseases. Now scientists are making increasing efforts to develop new treatments by targeting ubiquitin ligase or DUB, which directly or indirectly regulates the ER stress response.

## Figures and Tables

**Table 1:** E3 ubiquitin ligases and target proteins involved in the endoplasmic reticulum (ER) stress.

E3 ligase	Target	Impact on ER Stress	References
MITOL	IRE1α	Inhibits ER stress	[78]
CHIP	IRE1	Antagonizes ER stress-induced cellular senescence	[79]
HRD1	IRE1α	Inhibits ER stress	[80,81,82]
Parkin	CHOP	Inhibits ER stress	[82,83,84]
RNF183	Bcl-xL	Increases ER stress-induced apoptosis	[85]
RNF186	Bnip1	Increases ER stress-induced apoptosis	[86]
BAR	BI-1	Increases ER stress	[87,88,89]
GP78	?	Protects against ER stress and regulates lipid metabolism in *zebrafish* liver	[90]
GP78	HERP	Initiates the process of ER stress recovery	[91]
POSH	HERP	Maintains calcium homeostasis during ER stress	[92]

**Table 2:** DUBs and target proteins involved in ER stress.

DUB	Target	Impact on ER stress	References
ERAD			
USP13	Ubl4A	Promotes ERAD	[137]
USP19	TCRa and other ERAD substrates	Saves the substrate degradation caused by ERAD	[138]
	HRD1	Maintains normal ERAD process	[139]
YOD1	RI332 and other ERAD substrates	Promotes the reverse transport of misfolded proteins	[140,141]
**UPR**			
USP14	IRE1α	Inhibits ER stress-induced cell death	[142,143]
BAP1	CHOP/ATF3	Inhibits ER stress-induced apoptosis	[144]
USP5	ORP8	Increases ER stress	[145]
USP19	?	Inhibits the UPR reaction	[146]

## Data Availability

Not applicable.
